# Generate Analysis-Ready Data for Real-world Evidence: Tutorial for Harnessing Electronic Health Records With Advanced Informatic Technologies

**DOI:** 10.2196/45662

**Published:** 2023-05-25

**Authors:** Jue Hou, Rachel Zhao, Jessica Gronsbell, Yucong Lin, Clara-Lea Bonzel, Qingyi Zeng, Sinian Zhang, Brett K Beaulieu-Jones, Griffin M Weber, Thomas Jemielita, Shuyan Sabrina Wan, Chuan Hong, Tianrun Cai, Jun Wen, Vidul Ayakulangara Panickan, Kai-Li Liaw, Katherine Liao, Tianxi Cai

**Affiliations:** 1 Division of Biostatistics School of Public Health University of Minnesota Minneapolis, MN United States; 2 Department of Medicine University of British Columbia Vancouver, BC Canada; 3 Department of Statistical Sciences University of Toronto Toronto, ON Canada; 4 Institute of Engineering Medicine Beijing Institute of Technology Beijing China; 5 Department of Biomedical Informatics Harvard Medical School Boston, MA United States; 6 School of Statistics Renmin University of China Bejing China; 7 Department of Medicine University of Chicago Chicago, IL United States; 8 Merck & Co, Inc Rahway, NJ United States; 9 Department of Biostatistics & Bioinformatics Duke University Durham, NC United States; 10 Division of Rheumatology, Inflammation, and Immunity Department of Medicine Brigham and Women’s Hospital, Harvard Medical School Boston, MA United States; 11 Department of Biostatistics Harvard TH Chan School of Public Health Boston, MA United States

**Keywords:** electronic health records, real-world evidence, data curation, medical informatics, randomized controlled trials, reproducibility

## Abstract

Although randomized controlled trials (RCTs) are the gold standard for establishing the efficacy and safety of a medical treatment, real-world evidence (RWE) generated from real-world data has been vital in postapproval monitoring and is being promoted for the regulatory process of experimental therapies. An emerging source of real-world data is electronic health records (EHRs), which contain detailed information on patient care in both structured (eg, diagnosis codes) and unstructured (eg, clinical notes and images) forms. Despite the granularity of the data available in EHRs, the critical variables required to reliably assess the relationship between a treatment and clinical outcome are challenging to extract. To address this fundamental challenge and accelerate the reliable use of EHRs for RWE, we introduce an integrated data curation and modeling pipeline consisting of 4 modules that leverage recent advances in natural language processing, computational phenotyping, and causal modeling techniques with noisy data. Module 1 consists of techniques for data harmonization. We use natural language processing to recognize clinical variables from RCT design documents and map the extracted variables to EHR features with description matching and knowledge networks. Module 2 then develops techniques for cohort construction using advanced phenotyping algorithms to both identify patients with diseases of interest and define the treatment arms. Module 3 introduces methods for variable curation, including a list of existing tools to extract baseline variables from different sources (eg, codified, free text, and medical imaging) and end points of various types (eg, death, binary, temporal, and numerical). Finally, module 4 presents validation and robust modeling methods, and we propose a strategy to create gold-standard labels for EHR variables of interest to validate data curation quality and perform subsequent causal modeling for RWE. In addition to the workflow proposed in our pipeline, we also develop a reporting guideline for RWE that covers the necessary information to facilitate transparent reporting and reproducibility of results. Moreover, our pipeline is highly data driven, enhancing study data with a rich variety of publicly available information and knowledge sources. We also showcase our pipeline and provide guidance on the deployment of relevant tools by revisiting the emulation of the Clinical Outcomes of Surgical Therapy Study Group Trial on laparoscopy-assisted colectomy versus open colectomy in patients with early-stage colon cancer. We also draw on existing literature on EHR emulation of RCTs together with our own studies with the Mass General Brigham EHR.

## Introduction

Transforming real-world data (RWD) to real-world evidence (RWE) has the potential to augment the clinical knowledge gained from trial findings [1]. RWD offers a rich variety of clinical data from a broad patient population that are often infeasible to collect in traditional randomized controlled trials (RCTs). Thus, RWE generated from a large population is positioned to address questions of treatment effects across subgroups where RCTs are often underpowered, infeasible, or unethical [2-5]. In contrast to RCTs, which are designed to answer a specific question regarding the effectiveness of an intervention, many types of RWD are not structured for research. For example, electronic health records (EHRs) are primarily generated for clinical care and billing purposes, where useful clinical information may be dispersed among large volumes of data. Thus, to effectively use RWD, the data curation process and data quality must be critically evaluated before generating RWE for regulatory purposes [6].

The Food and Drug Administration defines RWD as data related to patient health status or delivery of health care, such as administrative claims, EHRs, and clinical or product registries [7]. RWE is defined as the clinical evidence regarding the use, benefits, or risks of a medical treatment derived from RWD [7]. To accelerate the use of RWE in the “discovery, development and delivery” of medical treatments, the 21st Century Cures Act and the subsequent Food and Drug Administration RWE framework laid the groundwork for the use of RWD in regulatory decision-making, including approvals for new indications of approved drugs and postapproval requirements [7-9].

EHRs have emerged as a primary source of RWD but present considerable challenges in data quality and statistical analysis for comparative effectiveness studies [10,11]. The release of “Meaningful Use” criteria by the Department of Health and Human Services greatly accelerated the adoption of EHRs among providers [12,13]. Through programs such as Common Data Models and Research Patient Data Repository, the structured data formats in EHRs have become increasingly standardized across health care systems and providers [14-16]. On the basis of these efforts, most existing RWE studies focused on the use of structured EHR features. Bartlett et al [11] investigated the feasibility of RCT emulation with both EHRs and insurance claims and identified the lack of critical data as the major limitation. Among 220 RCTs, 85% (187/220) were deemed infeasible for replication with EHR data because of the lack of readily usable structured data on (1) the inclusion and exclusion criteria, (2) the intervention, (3) the indication, or (4) the primary end point. However, this evaluation was based solely on structured data, such as the International Classification of Diseases (ICD) and current procedural terminology (CPT) codes, which do not fully capture information on phenotypes, procedural interventions, indication qualifiers, imaging results, and functional disease scores required for RCTs [11]. Although a few reporting guidelines regarding code-variable mapping and time windows have been proposed to improve the transparency and reproducibility of RWE [17,18], no clear data curation or statistical analysis guidelines have been developed. Harnessing unstructured data, such as clinical notes and images, can provide a more granular view of a patient’s health status that is not captured in structured data and can expand the availability of critical data for RWE generation. The common practice of using only structured EHR features mapped to clinical variables through description matching has been reported to have data quality issues and is inferior to mining unstructured data using advanced techniques [19].

Although EHRs have significant potential to generate RWE, the advances in medical informatics required to effectively leverage the rich information in both structured and unstructured data have not been widely adopted in the RWE community. In recent years, natural language processing (NLP) tools have been developed to extract information from various clinical notes including signs and symptoms, laboratory test values, and tumor progression. In addition, artificial intelligence (AI) has been successful in medical imaging (eg, computed tomography and magnetic resonance imaging) classification [20,21], segmentation (locating the region of interest) [22,23], and registration (merging information from multiple images) [24,25]. Despite these advancements in data curation technologies, there is still a need for approaches to efficiently extract clinical information that cannot be conveniently identified by codified EHR features, such as cancer metastasis status.

Phenotyping methods that combine multiple EHR features have been developed to improve the accuracy of disease status or outcome definitions, with the goal of creating a cohort of individuals with phenotypes for downstream studies. Advanced machine learning methods for phenotyping are now available to accurately and efficiently identify patients with specific medical conditions and clinical characteristics based on the comprehensive information extracted from their EHR and the temporal information of clinical events [26]. These technologies can enable the reliable extraction of EHR data to generate RWE. However, the existing methods are typically published in technical journals that are unfamiliar to most medical researchers. Moreover, deploying NLP, AI, and machine learning methods requires substantial expertise and guidance beyond what is typically available in most published studies and open-source software. Therefore, it is critical to establish a standard for presenting deployed data mining methods in a transparent manner that enables external validation of their performance. Finally, subsequent analyses should incorporate robust statistical methods to minimize the bias from imperfect data and confounders.

**Figure 1:**
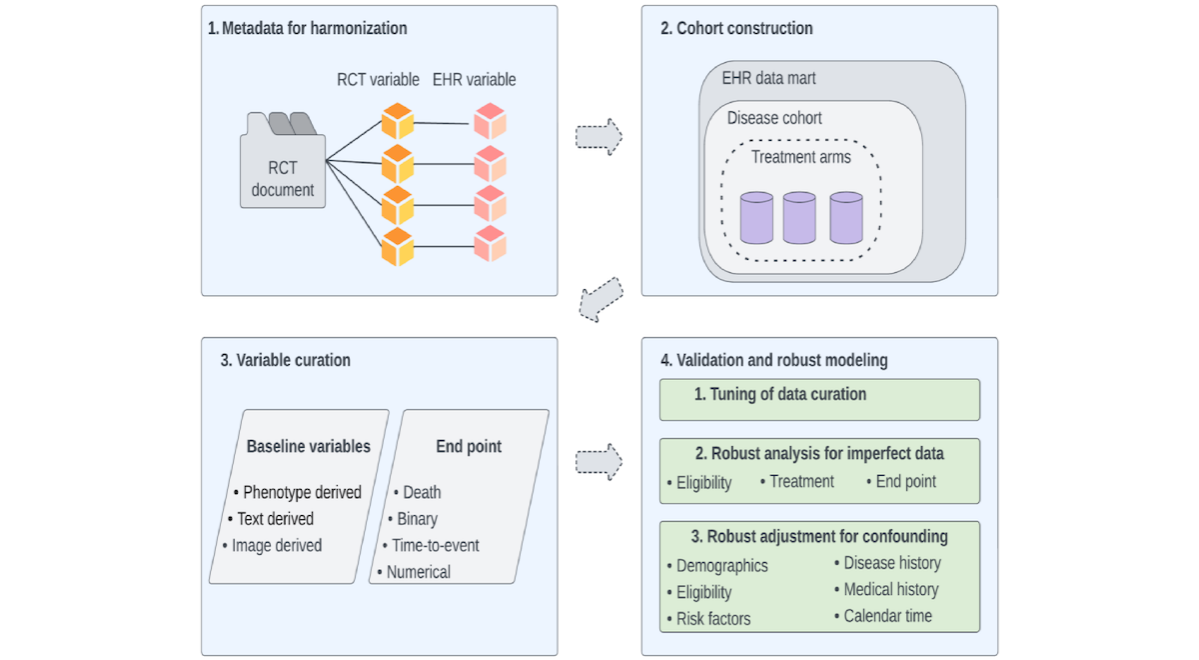
The integrated data curation pipeline designed to enable researchers to extract high-quality data from electronic health records for real-world evidence. EHR: electronic health record; RCT: randomized controlled trial.

In this paper, we propose an integrated pipeline to improve the resolution of EHR data for precision medicine research, bridging the gap between technological innovation and application to clinical studies. The pipeline addresses the unmet needs in RWE generation by streamlining the curation of previously unavailable variables and quality assurance steps, with an emphasis on the transparency and reproducibility of the data creation process. By incorporating new informatics tools and statistical methods developed over the past 5 years, we summarize the technologies and methods available for data curation and causal modeling that will enable researchers to perform robust analysis with high-resolution data from EHRs for RWE generation. Our pipeline has four modules as follows: (1) creating metadata for harmonization, (2) cohort construction, (3) variable curation, and (4) validation and robust modeling (Figure 1). Compared with existing practice in the RWE literature [27], our framework has 2 major advantages. First, we expand the availability of clinical variables by applying new technologies to unstructured data sources in *modules 1-3.* In *module 4,* we provide double assurance on the data quality with a validation against gold-standard annotations and a robust statistical analysis insensitive to data errors. To illustrate the application of the pipeline, we revisit the emulation of the clinical outcomes of surgical therapy (COST) Study Group Trial on laparoscopy-assisted colectomy versus open colectomy for patients with early-stage colon cancer as a running example [28]. We provide a brief description of the use case in each module, with expanded details in Multimedia Appendix 1 [28-44]. As 1 example cannot possibly cover all tools integrated into the pipeline, we create a repository with links for paper and codes of these tools organized according to the workflow of the pipeline [45]. A summary of the methods is provided in Table 1.

**Table 1:** Summary of methods in each step by their use.

Module	Step	Use	Methods
Data harmonization	Concept identification	Identify medical concepts from RCT^a^ documents	MetaMap [46], HPO^b^ [47], NILE^c^ [48], cTAKES^d^ [49]
Data harmonization	Concept matching	Grouping of structured EHR^e^	PheWAS^f^ catalog [32], CCS^g^ [50], RxNorm [51], and LOINC^h^ [52]
Data harmonization	Concept matching	Expansion and selection of relevant features using knowledge source or co-occurrence	Expert curation [33,53], knowledge sources [54-58], and EHR data [31,34,59-62]
Cohort construction	Data mart	Filter patients with diagnosis codes relevant to the disease of interest	PheWAS catalog [32] or HPO [47]
Cohort construction	Disease cohort	Identify patients with the disease of interest through phenotyping	Unsupervised: anchor and learn [63], XPRESS^i^ [64], APHRODITE^j^ [65], PheNorm [66], MAP^k^ [36], and sureLDA^l^ [67]; semisupervised: AFEP^m^ [57], SAFE^n^ [58], PSST^o^ [68], likelihood approach [69], and PheCAP [70]
Cohort construction	Indication and treatment arm	Identify indication conditions before treatment	Phenotyping with temporal input [37]
Variable curation	Extraction of baseline variables or end points	Extraction of binary variables through phenotyping	Phenotyping methods as listed out under cohort construction: disease cohort
Variable curation	Extraction of baseline variables or end points	Extraction of numerical variables through NLP^p^	EXTEND^q^ [71] and NICE^r^ [38]
Variable curation	Extraction of baseline variables	Extraction of radiological characteristics through medical AI^s^	For organs [72], blood vessels [73], neural systems [74,75], nodule detection [76,77], cancer staging [78], and fractional flow reserve [79,80]
Variable curation	Extraction of baseline end points	Extraction of event time through incidence phenotyping	Unsupervised [81,82], semisupervised [83,84], and supervised [85,86]
Downstream analysis	Causal inference for ATE^t^	Efficient and robust estimation of treatment effect with partially annotated noisy data	SMMAL^u^ [87]

^a^RCT: randomized controlled trial.

^b^HPO: human phenotype ontology.

^c^NILE: narrative information linear extraction.

^d^cTAKES: clinical text analysis and knowledge extraction system.

^e^EHR: electronic health record.

^f^PheWAS: phenome-wide association scans.

^g^CCS: clinical classification software.

^h^LOINC: logical observation identifier names and codes.

^i^XPRESS: extraction of phenotypes from records using silver standards.

^j^APHRODITE: automated phenotype routine for observational definition, identification, training and evaluation.

^k^MAP: multimodal automated phenotyping.

^l^sureLDA: surrogate-guided ensemble latent Dirichlet allocation.

^m^AFEP: automated feature extraction for phenotyping.

^n^SAFE: surrogate-assisted feature extraction.

^o^PSST: phenotyping through semisupervised tensor factorization.

^p^NLP: natural language processing.

^q^EXTEND: extraction of EMR numerical data.

^r^NICE: natural language processing interpreter for cancer extraction.

^s^AI: artificial intelligence.

^t^ATE: average treatment effect.

^u^SMMAL: semisupervised multiple machine learning.

### Integrated Data Curation and Modeling Pipeline for RWE

#### Overview

We begin by providing a high-level description of the related tools for each module of the pipeline. Next, we provide guidance on the deployment of the tools. Throughout this section, we frequently refer to *gold-standard labels* as the value or definition of a clinical variable curated by domain experts through a manual review of the EHR of selected patients.

#### Running Example

The COST Study Group Trial assessing laparoscopy-assisted colectomy versus open colectomy in the overall survival of patients with early-stage colon cancer was chosen as the target trial [29,30]. The inclusion criteria were as follows: a clinical diagnosis of adenocarcinoma of the colon, age of at least 18 years, and the absence of prohibitive abdominal adhesions. The exclusion criteria were as follows: advanced local or metastatic disease, rectal or transverse colon cancer, acute bowel obstruction or perforation from cancer, and severe medical illness. Inflammatory bowel disease, familial polyposis, pregnancy, or concurrent or previous malignant tumor also precluded enrollment. We showcased the pipeline by emulating the RCT using EHR data up to January 1, 2020, from Mass General Brigham [28].

#### Module 1: Creating Metadata for Harmonization

##### Background

Generating RWD relevant to a target RCT from EHR requires first curating EHR features corresponding to indication, intervention, end point, eligibility criteria, and patient characteristics considered in the trial. Unfortunately, many clinical variables involved in RCTs are not readily available in EHRs. The first step of our pipeline, *data harmonization* between the RCT study and EHR data, maps the clinical variables of interest to 1 or more relevant sources of EHR data. Our work is built on previous efforts to standardize structured EHR data [14-16] but also combines advancements in NLP and medical knowledge networks. The actual *extraction of clinical variables* is described in *module 3*, in which potential discrepancies among multiple sources are reconciled. In existing RWE studies, exact details of how the mapping was performed are rarely reported and often cannot be easily transported to another EHR system. Domain experts might have manually mapped inclusion or exclusion criteria, for example, if a patient was on a specific treatment, which can be labor intensive. The proposed procedure leverages NLP to improve the efficiency and transparency of the mapping process, making it scalable and portable for data harmonization.

We recommend the creation of the metadata needed for data harmonization by the following 2 key steps.

##### Concept Identification

Identify the medical concepts associated with the clinical variables from the RCT documents. This can be achieved by converting relevant textual information regarding clinical variables within the RCT documentation to established medical concepts using existing clinical NLP tools such as MetaMap, NILE (narrative information linear extraction), and cTAKES (clinical text analysis and knowledge extraction system) [46,48,49].

A medical concept can be represented by different names, for example, “RA” and “rheumatoid arthritis” are used to refer to the same medical concept—rheumatoid arthritis. The unified medical language system (UMLS) [88] maintained by the National Library of Medicine is a collection of biomedical vocabularies that maps a concept to all the names from all the source vocabularies that mean the same thing. The latest release of the UMLS consists of over 4 million concepts that are each represented by a concept unique identifier (CUI). For example, both “rheumatoid arthritis” and the abbreviation “RA” are mapped to the same CUI, C0003873. Different expressions for a low fever, such as “low grade fever” and “mild pyrexia,” are mapped to the same CUI, C0239574.

Using this map between concepts and terms, named entity recognition, can identify medical concepts in the text extracted from EHR, such as diseases, conditions, signs and symptoms, or medications. Named entity recognition is available in many existing clinical NLP software that use concept mapping in the backend to identify the concepts that are relevant for the study [49]. The dictionary of relevant medical concepts is used as the input for the variable extraction step described in *module 3* of our pipeline.

##### Concept Matching

Match the identified medical concepts to both structured and unstructured EHR data elements.

The identified CUIs, along with negation flags, offer an immediate source for NLP features to be processed by machine learning algorithms in *modules 2 and 3*. For example, if the eligibility criterion includes patients with rheumatoid arthritis, an NLP feature that counts the total number of mentions of the corresponding CUI “C0003873” can be used as a mapped NLP feature. However, as the mentions of relevant clinical variables in unstructured text can be nonspecific, we recommend concept matching to match the identified medical concepts to associated structured EHR data, for example, ICD codes, whenever possible. Grouping similar “structured variables” is helpful because the relationships among structured EHR variables are not reflected in existing hierarchical coding systems. Hong et al [31] provided a standard way to group structured EHR, which produced the mapping dictionary from the group names. Specifically, 4 domains of codified data were considered: diagnosis, procedures, laboratory measurements, and medications. Clinical variables under any of the domains were matched to the corresponding group using a group name search. ICD codes were aggregated into phecodes to represent more general diagnoses, for example, “MI” rather than “acute MI of inferolateral wall,” using the ICD-to-phecode mapping from PheWAS (phenome-wide association scans) catalog [32]. Multiple levels of granularity of phecode, including integer level, 1-digit level, and 2-digit level, can be used depending on the disease of interest. A popular alternative is the human phenotype ontology (HPO) [47]. For procedure codes, including CPT-4 (CPT 4th Edition), HCPCS (Healthcare Common Procedure Coding System), ICD-9-CM (ICD, Ninth Revision, Clinical Modification) Procedure Codes, and ICD-10-PCS (ICD, 10th Revision, Procedure Coding System) (except for medication procedures), clinical classification software categories were assigned based on the clinical classification software mapping [50]. For medication codes, the prescription encodings in a single EHR system were aggregated to the ingredient level RxNorm codes, the normalized names for clinical drugs developed by the National Library of Medicine [51]. For laboratory measurements, laboratory order encodings were grouped into manually annotated laboratory concepts or logical observation identifier names and codes (LOINC) [52]. The 4 domains of the grouped structured EHR variables provide another part of the raw data for variable extraction.

It is important to note that some clinical variables, for example, cancer stage and cancer recurrence, are poorly represented by specific structured codes and cannot be mapped to structured data. For example, cancer recurrence and cancer progression are poorly structured in EHR despite their important role in conveying a patient’s status. We recommend expanding the mapping to lists of relevant variables. To learn the relevance of medical variables from expert curation [33,53], knowledge sources [54-58] (compiled from Wikipedia pages, journal papers, the Merck Manual, etc), or EHR data [31,34,59-62], existing studies have developed (1) dictionaries of relevant variables [57,58]; (2) knowledge graphs with variables as vortex and relevance as edge [33,53,89,90], in which neighboring vortexes of the target variable form the dictionary of relevant variables; and (3) semantic embeddings with angles reflecting relevance and length reflecting frequency [34,54-56,59-61], from which the dictionary of relevant variables is compiled with vectors of small cosine similarities to the target variable. In addition, extracting clinical variables from data requires tools that can directly access raw text and image reports. We describe the methods used to accommodate these settings in the variable extraction section (*module 3*).

##### Running Example: Emulation of COST Study Group Trial (Section S1)

We extracted sections 3.0 Patient Eligibility, 5.0 Stratification Factors from the study protocol [30] and the first paragraphs in the Methods and Results sections along with Table 1 from the reporting paper [29]. From the extracted text, we used NILE to identify the list of medical concepts [48]. Through an algorithm matching text medical concepts to feature or grouping descriptions [32,35], we obtained the list of EHR features relevant to the RCT design. The comparison with previously reported manual mapping [28] demonstrated the capability of our scalable harmonization (Table S1 in Multimedia Appendix 1).

#### Module 2: Cohort Construction

##### Overview

The construction of the study cohort for RWE involves identifying the patients with the condition or disease of interest (often referred to as the phenotype), their time window for the indication, and whether they underwent the interventions in the RCT. EHRs contain a large amount of data; however, only a subset is relevant to any given study. It is also necessary to simultaneously safeguard against the risk of inadvertent use, including unnecessary personal health identifiers when using the data for analysis. To enable accurate condition or disease identification while maintaining patient privacy, we recommend a 3-phase cohort construction strategy that extracts the minimally necessary data from the EHR, beginning with an overly inclusive data mart that is used to develop the disease cohort and then to establish the treatment arms.

##### Phase 1: Data Mart

The data mart is defined as the subset of patients from the entire data warehouse who potentially meet the relevant criteria for a study. In the context of generating RWE, we design the data mart to include all patients with any indication of the disease or condition of interest. To ensure inclusivity, researchers should summarize a broad list of EHR variables with high sensitivity and construct the data mart to capture patients with at least 1 occurrence of the listed variables. A typical choice is the disease-specific phecode. Most phecodes are sensitive for phenotypes of interest but are often nonspecific [70]. We recommend validating the inclusiveness of the broad list by obtaining a small set of gold-standard labels by reviewing patient charts for the presence or absence of the phenotype sampled in a case-control manner, for example, 20 patients selected from the data mart and 20 patients selected from outside the data mart. More details are provided in *module 4*. If the validation indicates that the data mart definition is not broad enough and patients with the phenotype are not identified, expansion to relevant variables may be developed using the existing resources described in *module 1*. Conversely, if the definition is overly inclusive so that many patients without the phenotype are captured, a narrower list can be constructed by going 1 level down in the PheWAS catalog hierarchy or using more specific ICD codes.

##### Phase 2: Disease Cohort

After the data mart is created, the next phase is to identify the disease cohort consisting of the subset of patients within the data mart who have the phenotype of interest. Identification of the disease cohort is referred to as phenotyping in the informatics literature and has been well studied over the last decade [26,91,92]. Commonly used phenotyping tools can be generally classified as either (1) expert guided (mostly rule based) or (2) derived from machine learning methods. Expert-guided approaches are simple to develop using clinical and informatics knowledge that can be translated into a set of rules based on EHR variables. However, expert-guided approaches are difficult to generalize across diseases and databases, as they must be constructed in a case-by-case manner [93-95]. Machine learning–based approaches are further classified as either weakly supervised, semisupervised, or supervised based on the availability of gold-standard labels for model training. A comprehensive review of this topic is presented by Yang et al [26]. Weakly supervised machine learning approaches have become increasingly popular because they are trained without gold-standard phenotype labels, which are time consuming to obtain. Instead, model training is based on the so-called *silver-standard labels*. Silver-standard labels are variables that can be readily extracted for all patients in the database but are imperfect measurements of the underlying phenotype (eg, associated phecodes or CUIs). Examples of existing weakly supervised approaches include the anchor-and-learn approach [63], extraction of phenotypes from records using silver standards (XPRESS) [64], automated phenotype routine for observational definition, identification, training and evaluation (APHRODITE) [65], PheNorm [66], multimodal automated phenotyping (MAP) [36], and surrogate-guided ensemble latent Dirichlet allocation (sureLDA) [67]. Alternatively, semisupervised approaches augment the silver-standard labels with a small set of gold-standard labels. These approaches are more time consuming than weakly supervised learning approaches because of the necessity of labeled data, but they can be more accurate when the silver-standard labels are poor measures of the underlying disease (eg, psychological or behavioral conditions). Common semisupervised approaches include AFEP (automated feature extraction for phenotyping) [57], SAFE (surrogate-assisted feature extraction) [58], PSST (phenotyping through semisupervised tensor factorization) [68], likelihood-based approaches [69], and PheCAP [70]. All these methods output probabilities of the disease for each patient, rather than a deterministic classification, which may be leveraged in subsequent modules. Although supervised approaches have decreased in popularity owing to their high demands for gold-standard labeled data [96,97], they may be applied to new or rare diseases without established silver-standard labels.

##### Phase 3: Treatment Arms and Timing

With the disease cohort, one may then proceed to determine which patients received the treatments relevant to the indication of interest. Most treatment information is well coded as part of the structured EHR data in the medication and procedure codes. For example, in-hospital procedures and medications are closely recorded using designated structured codes. The indication information, however, may require learning the temporal order of the treatment initiation and the disease onset or progression. For example, the first-line therapy for metastatic or recurrent cancer is defined by the pattern “metastasis or recurrence, then use of the chemotherapy before any other therapies” [98]. In such cases, it is necessary to ascertain both the treatment initiation time and the occurrence time of metastatic or recurrent cancer to ensure the correct temporal order. Phenotyping methods incorporating the temporal order of EHR variables [37] are suitable for identifying patients matching the indication. The treatment initiation time is then typically set as time zero in the study, which is later used for variable curation in module 3.

##### Running Example: Emulation of COST Study Group Trial (Section S2)

The data mart consists of 65,968 patients with diagnosis codes mapped to phecode 153 (colorectal cancer) [32]. Using the total occurrences of phecode 153, the total mentions of CUI C0009402 (colorectal cancer), and the number of days of medical encounters, we determined colorectal cancer status through MAP [36]. The MAP scores achieve a 0.945 area under the curve of receptor operating characteristics evaluated over 171 gold-standard labels. MAP>0.371 identified 28,859 patients as colorectal cancer cases (specificity=0.95; sensitivity=0.70; and positive predictive value=0.90). Colectomy and laparoscopy-assisted colectomy were then identified using procedure codes with descriptions containing “partial colectomy.” We refined the treatment arms to match the indications of the target RCT by obtaining the timings of the initial colorectal cancer diagnosis and other surgical procedures.

#### Module 3: Variable Curation

##### Overview

The emulation of RCT with EHR data generally requires three categories of data elements: (1) the end points measuring the treatment effect, (2) the eligibility criteria defined to match the RCT population, and (3) the confounding factors to correct for treatment-by-indication biases inherent to RWD. In this section, we describe the classification and extraction of the first 2 types before addressing the confounding factors in *module 4*. Our classification of variables is based on 3 rules: the format of the variable source (phenotype, text, or image), its structure in EHR (well or poorly structured), and the need to use phenotyping algorithms to improve its resolution. Well-structured variables have a clear mapping to organized EHR codes (eg, diseases listed in the PheWAS catalog), whereas poorly structured ones do not (eg, disease progression). Even for well-structured data elements, there may be a need to improve the accuracy of a clinical variable, such as the disease status discussed in *module 2*, owing to the noisiness of the EHR codes. We study the eligibility criteria and confounding factors together, as they are covered by the general pretreatment baseline variables.

##### Baseline Eligibility Criteria

The list of eligibility criteria is provided by the RCT protocol and mapped to the corresponding EHR variables in *module 1*. The list of variables available in the RCT data or reported in the corresponding paper can then be used by the user to perform population adjustment (eg, weighting or matching).

Baseline variables were classified into 3 types: *phenotype derived*, *text derived,* and *image derived. Phenotype*-*derived* variables have a clear correspondence with codified data, for example, the onset of disease or past use of a medication. The extraction of *phenotype*-*derived* variables is essentially performed by using a phenotyping algorithm, as discussed in *Module 2: Phase 2* section. If a variable is well structured, its EHR indicators may be used as silver-standard labels in unsupervised or semisupervised phenotyping methods. Otherwise, only supervised methods can be applied.

Extraction of the other 2 types of baseline variables may require specific tools. *Text*-*derived* variables include numerical data embedded in clinical notes with a tag such as a relevant concept or code in the vicinity. EXTEND (extraction of EMR numerical data) was developed to link the numbers to their tags and has been applied to BMI, ejection fraction, vital signs, and performance status (Eastern Cooperative Oncology Group or Karnofsky Performance Scale) with high accuracy [71]. A context-sensitive variant (NICE, NLP Interpreter for Cancer Extraction) was developed to disambiguate common features such as the stage of the disease of interest. NICE can also extract radiological or genetic information, for example, tumor size and mutation variant, from text reports along with a relevant date if the note points to a past event [38]. RCTs tend to adopt rigorous radiological evaluation criteria for eligibility, for example, diameter of cancer tumor in response evaluation criteria in solid tumors (RECIST) [39]. However, such evaluations were rarely measured and documented in real-world radiological reports, as reported in other studies [40]. With the advancement of image recognition technology, the extraction of *image*-*derived* evaluation from imaging data in EHR has become possible. Segmentation tools have been developed for organs [72], blood vessels [73], and neural systems [74,75], which may produce the physical measures. Diagnostic tools have been developed for nodule detection [76,77], cancer staging [78], and fractional flow reserve [79,80].

A preliminary emulation cohort can be constructed from the extracted eligibility criteria. Users may use a relaxed or conservative rule, depending on the anticipated sample size. In *module 4*, further modifications will be applied to finalize the emulation.

##### End Points

The extraction of end points varies depending on their type. We classify the end points into 4 categories: *death*, *binary*, *time-to-event,* and *numerical*. Death is singled out for its external source.

*Death* information can be obtained by linking EHR to national vital statistical databases. Caution should be exercised on possible data leakage or informative censoring, even for presumably reliable end points such as death. We noticed missing death status from patients with terminal-stage cancer, likely owing to out-of-state home hospice care. In this case, the end point should be modified to in-hospital death or discharge in a terminal condition. Discharge in terminal conditions can be extracted as typical binary phenotypes by semisupervised methods using EHR data from the last month before loss to follow-up.

*Binary* end points are essentially a binary status of the presence or absence of a clinical condition during or at the end of follow-ups, for example, 1-year remission of the disease. Therefore, they can be extracted by phenotyping methods using the EHR data since treatment initiation. As many end points consist of disease progression rather than diagnosis, they are poorly structured. Therefore, semisupervised phenotyping methods aggregating auxiliary information from other relevant features may be preferred to balance the resources needed to manually curate gold-standard labels via chart review and to accurately define the final end point.

*Time-to-event* end points include many common primary end points, for example, progression-free survival for cancer. The longitudinal trajectories of EHR features (eg, diagnosis and procedures) relevant to the event of interest provide information on the event time through incidence phenotyping. Incidence phenotyping can be tackled using various unsupervised [81,82], semisupervised [83,84], and supervised [85,86] approaches.

*Numerical* end points, including ordinal end points such as disease severity scores and real number end points such as tumor size, are usually difficult to extract from EHR. Tools for *text*-*derived* baseline variables provide an option for extraction, but missing documentation in the real-world setting imposes intrinsic difficulty. If a measurement is not captured at the specific time of interest, some temporal tolerance should be considered. Effort has been put into data-driven construction of severity scores from EHR for depression, multiple sclerosis, and stroke, in which a machine learning algorithm trained the EHR severity score on a labeled subset with standard severity scores derived from a registry, questionnaire, or NLP tool. For diagnosis-related baseline variables and end points, if there are no records on the diagnosis of interest, it typically indicates that the patient was never diagnosed with the condition, and as a result, it may be considered as a negative instance for the phenotype.

##### Missing Data

Missing data is a common issue for RWD. Some information may be absent in real-world medical records; thus, it is not even available for manual abstraction. For diagnosis-related baseline variables and end points, if there are no records on the diagnosis of interest, it typically indicates that the patient was never diagnosed with the condition, and as a result, it may be considered as a negative instance for the phenotype. For *text- or image*-*derived* baseline variables, *numerical* end points, or laboratory testing results, the absence of extraction should be marked as missing data. In downstream analyses, standard strategies can be used to handle missing data, imputation, or missing indicators. Caution should be exercised when dealing with potential informative missingness. If the missing rate is too high, compromise must be considered for the missing variables such as discarding from baseline variables or finding surrogates for end points. Sensitivity analyses can be performed to ensure that the results are consistent across the different strategies for handling missingness.

##### Running Example: Emulation of COST Study Group Trial (Section S3)

We extracted the overall survival end points from the linked death registry. To capture unreported death in the registry, we constructed a score for treatment termination in the terminal condition based on diagnosis and procedure codes in the last month of EHR encounters. Most baseline variables are *phenotype derived*; therefore, we extracted them through phenotyping method–based mapping from module 1. We extracted the cancer stage data through NICE [38]. We reported the list of variables along with the extraction methods in Section S3 in Multimedia Appendix 1.

#### Module 4: Validation and Robust Modeling

##### Overview

Inaccurate data curation and confounding can lead to biased RWE. Even with reasonably accurate medical informatics tools at disposal, remaining errors from data curation will be carried over to downstream analyses, potentially causing bias in treatment assessment. Confounding is a constant challenge in assessing treatment with observational data [99], including the routinely collected EHR. Confounding factors, variables that affect both the treatment assignment and outcome, must be properly adjusted. To minimize bias, the pipeline should include (1) validation for optimizing the medical informatics tools in *modules 2 and 3,* (2) robust statistical methods that produce consistent estimation of treatment effect from imperfect data [87,100,101], and (3) comprehensive confounding adjustment [28,102,103].

##### Validation and Tuning of Data Curation

First, we suggest validating the quality of data curation by detecting any inconsistency between annotation and extraction. When the validation of all variables is infeasible, priority should be given to variables defining the following : (1) indication and eligibility, (2) treatment arms, (3) end points, and (4) key confounding variables. To ensure a sufficient detection chance, we recommend the validation sample size formula (refer to Section S4 in Multimedia Appendix 1 for derivation): *Validation size ≥ log(1 − detection chance)/log(1 − error tolerance).*

Users can choose the detection chance and error tolerance according to the context and report these parameters along with validation results. With a 95% detection chance and 5% error tolerance, a subset of at least 59 is required. The validation set can be used for tuning the data curation when excessive error is detected. To avoid overfitting, we recommend using 2 validation sets, one for tuning and the other for posttuning revalidation.

##### Robust Analysis for Imperfect Data

Second, three annotations should be created for cohort emulation and robust downstream analysis:

1. Indicator for indication, arm, and eligibility. In addition to the levels for the treatment of interest, a level of exclusion should be created for patients who are not eligible.
2. Actual end points consistent with any modification as in *module 3*.
3. Other variables for population adjustments.

The size of this subset should be determined by the recommended sample size of the supervised or semisupervised methods used in downstream analysis. Annotations for variables with validation errors are created for this larger set. We describe a sampling scheme that efficiently recycles the annotation in Section S4 in Multimedia Appendix 1.

##### Robust Adjustment for Confounding

The list of confounding factors, however, is seldom known a priori. A common strategy in RWD treatment effect analysis is to include many probable confounding factors and capture the confounding with model selection techniques [28,102]. Here, we provide a comprehensive list for identifying potential confounding factors:

- *Demographic* data of the RCT are routinely described in the paper reporting the results of the target RCT. A list can be pulled from there.
- Some *eligibility criteria–* defining variables may have multiple eligibility levels or values. They usually carry clinical importance and are thus likely to affect both treatment and outcome in real-world practice.
- General *medical history* is described by the disease and symptom diagnoses, which include comorbidities. The diagnosis codes at baseline grouped into integer-level phecodes can be used.
- *Disease history* includes the disease severity, course of progression, and past treatments. Both an expert-defined approach and data-driven approach can be considered. The expert-defined list may come from a domain expert or the existing literature on related observational studies. The data-driven list can be generated through dictionaries, knowledge graphs and semantic embeddings similar to the mapping of poorly structured data in *module 1*.
- *Risk factors*, variables affecting outcomes, contain all confounders. A review of the literature on the disease will provide a list of the identified risk factors.
- *Calendar year of treatment initiation*: if the treatment initiation times in EHR cover a long time span or landmark change in practice, the calendar year may become the confounding factor [28].

Validation may not be necessary for the large number of potential confounding EHR factors because they are sufficient for explaining confounding even if they deviate from the apparent description. In the downstream analysis, we recommend the doubly robust estimation that produces an accurate treatment assessment if either the mechanism of treatment assignment (propensity scores) or outcome (outcome regression) is properly modeled [104].

##### Running Example: Emulation of COST Study Group Trial (Section S4)

We described the strategy to determine the validation sample size and sample the validation set. To account for confounding factors, we adjusted for clinically relevant variables such as age, sex, cancer stage, tumor location, colon adhesion, procedure subtypes, obesity, and a broad range of other comorbidities. We adopted a doubly robust causal modeling strategy [104] that combines (1) the regression adjustment approach via outcome regression and (2) the propensity score weighting approach. To account for temporal changes, we allowed the covariate effects in both the outcome regression and propensity score models to vary across the temporal periods but adopted a data-driven cotraining strategy to select temporal trends as well as confounding factors [41-43].

##### Guideline for Prespecification and Reporting

The creation of analysis-ready data plays an indispensable role in generating RWE from EHRs, which is evident from its substantial representation in our pipeline from harmonization to validation. Discrepancies in the data creation process may hinder the replication of RWE studies and the investigation of generalizability and transferability. On the basis of the components of the pipeline, we propose the guidelines for the prespecification and reporting of data creation (Table 2). The guidelines will supplement existing efforts advocating for a transparent, prespecified statistical analysis plan [105] to promote transparency and reproducibility of RWE.

**Table 2:** Guidelines for prespecification and reporting of data creation.

		Item number	Recommendation
**Data harmonization**			
	Target RCT^a^ study design	1	Prespecification: Indicate the source of the study design document (protocol, reporting paper, or others). Reporting: Describe the sections and tables from which the relevant variables are recognized.
	RCT variable list	2	Prespecification: Specify the method for recognizing variables from the RCT document and matching to relevant EHR^b^ features. Reporting: Define all end points, interventions, eligibility criteria, and other baseline characteristics recognized from the RCT document along with the matched EHR features.
**Cohort construction**			
	Data mart	3	Prespecification: Specify the method for compiling the broad list of EHR features indicating the condition or disease of interest. Reporting: List the EHR features, and state the algorithm to define inclusion in the data mart. Reporting: Report the size of the data mart.
	Disease cohort	4	Prespecification: Specify the method for ascertaining the phenotype of the condition or disease of interest. Reporting: Describe the input EHR features of the phenotyping algorithm. Reporting: State the phenotyping algorithm with chosen parameters. Reporting: Report the AUC^c^ of prediction and the accuracy of the disease cohort.
	Treatment arms	5	Reporting: Explain how treatment initiation time is determined. Reporting: Explain how treatment arms are defined with the list of involved EHR features, time windows, and the algorithm.
**Variable curation**			
	End points	6	Prespecification: Specify the method for ascertaining the end points. Reporting: State the end point algorithm with chosen parameters. Reporting: Explain how the end point is defined.
	Baseline characteristics (eligibility criteria and confounders)	7	Prespecification: Specify the variable curation plans for each class of baseline characteristics. Reporting: List the baseline characteristics considered in the RWE^d^ and define how they are created with input EHR features, time windows, groupings, and other transformation. Reporting: Explain how eligibility criteria will be matched according to the curated baseline characteristics. Reporting: Present the summary statistics of the baseline characteristics in treatment arms filtered by eligibility criteria.
	Additional confounders	8	Prespecification: List the other confounders considered in the RWE and define how they are created with input EHR features, time windows, groupings, and other transformation.
	Missing data	9	Reporting: Describe how missing information on variables are handled.
**Validation**			
	Sampling strategy	10	Prespecification: Specify the sampling strategy for the validation set. Reporting: Report the sizes and list of variables (in data mart, disease cohort, and arms) of the validation.
	Data accuracy	11	Reporting: Report the agreement between gold-standard data from validation and curated data. Reporting: Explain how inaccurate data are dealt with.
Publication		12	Reporting: Export the final curation models for the condition or disease of interest, end points, and other variables curated through machine learning methods.

^a^RCT: randomized controlled trial.

^b^EHR: electronic health record.

^c^AUC: area under the curve.

^d^RWE: real-world evidence.

## Discussion

### Summary

The data curation and modeling pipeline described in the paper demonstrates the wide-ranging potential applications of RWD in clinical development. For instance, RWD can be used to derive external or hybrid control arms or to conduct pragmatic trials. In the former case, the external control arm can serve as a benchmark for a single-arm design or can be used to augment an existing RCT control arm to improve study bias. Specifically, the proposed pipeline could (1) better identify patients who meet the target trial eligibility criteria along with an assessment of any discrepancy, (2) encourage harmonization between RCT and RWD variables to allow for easier statistical adjustments, (3) address missing data issues prevalent in RWD through efficient imputation strategies, and (4) extract more relevant variables by leveraging both structured and unstructured data. Overall, our pipeline aims to develop a fit-for-purpose RWD data set through robust and transparent data processing. This pipeline can also be used to generate RWD for other purposes. For instance, the RWD generated from EHRs can be used to expand or update existing observational study cohorts, thereby increasing the usability of RWD for applications such as safety. Although RWD may not always be suitable for a specific study of interest, our pipeline provides a roadmap for formatting RWD that can generate RWE available for downstream applications that can accelerate clinical development, ultimately leading to better patient care.

### Extension to Digital Twins

RWD is also recognized as a foundation for the creation of digital twins [106], an emerging concept borrowed from engineering to health care, which involves the creation of a health care data–based replica of patient data collected from digital technologies. This technique aims to improve precision health care by modeling and forecasting outcomes under available interventions based on data collected from digital technologies [107], which are increasingly integrated into EHRs [108]. Visionaries of digital twins advocate for automated data processing by AI, given the anticipated complexity of future digital health care data and the need for real-time decision-making. The notation of digital twins has strong resemblance with established concepts in causal inference, such as potential outcomes [109] and virtual twins [110]. In essence, digital twins in the precision health care setting will enable personalized optimization of interventions according to their forecasted outcomes derived from modeling of the outcome mechanism [107], which is a by-product of the robust causal modeling. The RWD generated by our data curation pipeline can also be used to form digital twins, complementing the existing precision medicine studies that relied on RCT data [111]. For clinical development in general, this can further improve the understanding of treatment heterogeneity and inform the study design.

### Conditions for Deploying the Data Curation Pipeline

To deploy the pipeline, certain conditions must be met. First, the EHR infrastructure should allow for the mapping of local codes to common ontologies for structured data such as ICD, CPT, and RxNorm. Second, the available medical notes and imaging data must provide sufficient information for medical experts in the research team to annotate the key clinical variables, ensuring the capture of the most routinely collected clinical information by EHRs. Notably, some variables intended for specific clinical trials, such as performance status, may not be universally available. Data from a single institution may not capture all relevant clinical information owing to well-known data leakage issues in patients who receive care at multiple centers or routinely take nonprescription medications [5,112]. Finally, the scalable extraction tools should have reasonable performance for the key clinical variables. Otherwise, no additional information can be obtained from the annotated subset.

### Limitations

For clinical development applications such as deriving external control arms, comparable with existing guidance for traditional RCTs, RWD-related statistical analysis plans should be prespecified and discussed with relevant regulatory agencies. Similarly, data curation plans should also be prespecified to ensure the reliability (data accuracy, completeness, provenance, and traceability) and relevance for supporting regulatory decisions. The proposed data curation process addresses these requirements by using a scalable framework for phenotyping and variable or outcome extraction. However, the following limitations of the scalable data curation process should be considered: (1) treatments that are relevant to RCTs but not typically administered in routine clinical practice, such as preapproval treatments and placebo, can be unavailable in RWD. (2) It is generally difficult to emulate RCT comparing an effective novel therapy with clearly inferior treatments owing to the treatment-by-indication bias. (3) Medication dose and regimen administration patterns can be inadequately documented in EHRs, making it challenging to emulate RCTs comparing doses or administration patterns of the same medication. (4) It may be difficult to extract certain RCT-specific clinical outcomes of interest, such as RECIST, from EHRs because they are not widely documented in routine patient care. (5) RWD documenting discrete medical encounters may not always precisely capture the temporal information of medical events that occur between visits. (6) Patients may undergo transfer between different health care systems, leading to potential disruptions in their treatment and incomplete capture of clinical end point information. (7) Imperfect extraction of key variables, such as confounding variables and clinical inclusion or exclusion criteria, can induce population or confounding biases. Potential solutions for part of the limitations include the following: (1) to consider alternative EHR metrics for RCT-specific clinical outcomes if they can be validated. (2) analysis of interval censoring data should be considered to characterize events between visits. (3) data consortium can be established across different health care systems may reduce data leakage. (4) domain experts can identify the crucial variables for a given study and aid in validation to minimize the bias from imperfect key variables.

## Appendix

Multimedia Appendix 1 Expanded details for methods and the running example.

## Abbreviations

AFEP: automated feature extraction for phenotyping
AI: artificial intelligence
APHRODITE: automated phenotype routine for observational definition, identification, training and evaluation
COST: clinical outcomes of surgical therapy
CPT: current procedural terminology
cTAKES: clinical text analysis and knowledge extraction system
CUI: concept unique identifier
EHR: electronic health record
EXTEND: extraction of EMR numerical data
HCPCS: Healthcare Common Procedure Coding System
HPO: human phenotype ontology
ICD: International Classification of Diseases
LOINC: logical observation identifier names and codes
MAP: multimodal automated phenotyping
NICE: natural language processing interpreter for cancer extraction
NILE: narrative information linear extraction
NLP: natural language processing
PSST: phenotyping through semisupervised tensor factorization
RCT: randomized controlled trial
RECIST: response evaluation criteria in solid tumors
RWD: real-world data
RWE: real-world evidence
SAFE: surrogate-assisted feature extraction
sureLDA: surrogate-guided ensemble latent Dirichlet allocation
UMLS: unified medical language system
XPRESS: extraction of phenotypes from records using silver standards

## Data Availability

The data sets generated or analyzed during this study are not publicly available because of regulations on protected health information but are available from the corresponding author on reasonable request.
