# Supplementary material for: Generate Analysis-Ready Data for Real-world Evidence: Tutorial for Harnessing Electronic Health Records With Advanced Informatic Technologies
Source: J Med Internet Res. 2023 May 25;25:e45662. doi: 10.2196/45662 (PMC10251230; doi:10.2196/45662)
Supplement: Multimedia Appendix 1 [file jmir_v25i1e45662_app1.pdf]

Multimedia Appendix

Section S1. Details and running example for Module 1…………………………………………… 2

- **Table S1.** Mapping from COST Study Group Trial variables to EHR features. ...……… 4
- **Table S2.** Mapping between EHR feature types and UMLS semantic types. ..………… 4

Section S2. Details and running example for Module 2. ……………………………………….. 5

Section S3. Details and running example for Module 3. …………………………………………9

Section S4. Details and running example for Module 4. …………………………………….....13

Reference in Multimedia Appendix………………………………………………………………...15

Section S1. Details and example for Module 1.

We conducted Concept Identification and Concept Matching using Java and R programs, respectively to analyze and process medical data. We created free-text description of the Clinical Outcomes of Surgical Therapy (COST) Study Group Trial by processing the reporting protocol [30] and paper [29] in pdf format with java packages PDDocument and PDFTextStripper.

Concept Identification: We identified the clinical concepts relevant to randomized clinical trial (RCT) design, which are represented by unique concept identifiers (CUIs) in the Unified Medical Language System (UMLS) [33]. We compiled a dictionary from UMLS medical terms to CUIs dictionary through a filter on informative semantic types (**Table S2**). Processing the free-text trial paper with the filtered CUI dictionary through NILE [48], we obtained the list of clinical concepts relevant to the trial design and analysis (**Table S1**).

Concept Matching: We matched the list of recognized clinical concepts to both structured Electronic Health Record (EHR) data elements. For COST Group Trial, most recognized clinical concepts belonged to the diagnosis or the procedure category. We performed partial string matching between all terms of the recognized CUIs and the descriptions of the EHR features/groupings. For diagnosis, we considered the ICD-9, ICD-10, ICD-10-CM diagnosis codes and their PheCode groupings [32]. For procedures, we considered ICD-9, ICD-10 procedure codes, current procedure terminology (CPT) codes and their groupings in Clinical Classification Software (CCS) [35]. We measured the specificity of the matched descriptions by the proportion of searching terms in terms of word counts. For example, the description “computerized axial tomography (ct) scan” has a 0.6 score for searching term “computerized axial tomography” from CUI C0040405 (3 out of 5 words). We applied the following rules in cleaning the mapping.

List of nonspecific terms such as “disease”, “acute” or any terms with 3 letters or less were excluded from the dictionary.

1. Only features with description achieving at least 0.4 score were kept.
2. Matching to groupings (PheCode and CCS) were preferred over matching to base codes (ICD-9, ICD-10, CPT). If a concept is matched to a grouping and some other base codes, we will keep the grouping along with any base code of strictly higher score than the grouping.
3. Matching from parent terms were preferred over that from child terms. For example, we may recognize parent term “acute appendicitis” C0085693 along with child term “appendicitis” C4553526. If matching of the parent term is successful, none of its child terms will be considered.
4. For concepts without a direct mapping, we used the KESER knowledge network to find codes with top cosine similarities[31]. A shiny interface is available at https://dev.parse-health.org/shiny/ARCH/.

The final mapping was presented in **Table S1**. Compared with the expert guided approach in the prior emulation of COST Study Group Trial [28], the scalable approach recovered mapping of key eligibility criteria on colon cancer, concurrent cancer, transverse colon cancer, rectal cancer, Crohn disease, bowel obstruction, metastasis familial polyposis and perforated colon to structured codes. In addition, the scalable approach suggested a list of CUIs as potential alternatives to structured data. The identification of treatment procedures and tumor locations, however, requires further curation in Modules 2 and 3.

Table S1. Mapping from COST Study Group Trial variables to EHR features. *Italic codes* indicate mapping from KESER network.

Description	CUI	Semantic	Codes
**Eligibility**			
Neoplasms/cancer	C0027651	neoplastic process	PheCode Class: 140-165, 170-199, 200-204, 208-230, 289, 610-611, 796, 860
Transverse colon cancer	C0153434	neoplastic process	ICD-9: 153.1; ICD-10: C18.4
Bowel obstruction	C0021843	disease or syndrome	ICD-9: 560.9; ICD-10: K56.60
Rectal cancer	C0949022	neoplastic process	ICD-9: 154.1; ICD-10: C20
Crohn disease	C0010346	disease or syndrome	ICD-9: 555; ICD-10: K50.0, K50.1, K50.9
carcinoma of the colon	C0699790	neoplastic process	*PheCode:153*
metastatic disease	C0027627	neoplastic process	*PheCode:198*
familial polyposis	C0032580	neoplastic process	*PheCode:211*
perforated colon	C0347646	disease or syndrome	*PheCode:853*
**Intervention**			
open colectomy	C0149750	therapeutic or preventive procedure	*CCS: 66, 153*
colectomy	C0009274	therapeutic or preventive procedure	*CCS: 66, 153*
laparoscopic procedure	C0521291	diagnostic procedure	*CCS: 66, 153*
**Confounding**			
Chemotherapy	C0392920	therapeutic or preventive procedure	PheCode: 197; CCS: 224.
Adhesions	C0001511	pathologic function	*PheCode:560.3, 568.1*

Table S2. Mapping between EHR feature types and UMLS semantic types.

EHR Feature Types	UMLS Semantic Types
Diagnosis	disease or syndrome, sign or symptom, neoplastic process, organ or tissue function, pathologic function.
Procedure	therapeutic or preventive procedure, diagnostic procedure
Laboratory tests	laboratory procedure, laboratory or test result
Medication	pharmacologic substance, biologically active substance, hormone, amino acid, peptide, or protein, organic chemical.

Section S2. Details and example for Module 2.

Module 2 (cohort construction) is generally similar across studies. We provide a step by step description of its application on the emulation of COST Study Group Trial.

<u>Build the data mart.</u> Map the disease to its associated PheCode. Extract the patients with the diagnosis code under the PheCode from database. For example, to create a data mart for colorectal cancer, all subjects with PheCode 153 for colorectal cancer would be included. The list of diagnosis codes includes:

1. ICD-9: 153.x, 154.x, 159.0, 209.1x, 230.3-6, 796.7x, V10.05-06.
2. ICD-10-CM: C18.x, C19, C20, C21.0-2, C21.8, C26.0, D01.0-3.
3. ICD-10-CM: C7A.020-025, C7A.029, R85.610-614, R85.619, Z85.03, Z85.030, Z85.038, Z85.04, Z85.040, Z85.048.

<u>Construct the disease cohort.</u> Many phenotyping algorithms require the silver-standard labels, often the total counts of associated PheCodes or CUIs. Some also require a feature that is a proxy for healthcare utilization to account for the heterogeneity in the dataset. A set of gold-standard labels should be generated for validating the performance of the phenotyping algorithm. Additional gold-standard labels are needed for training supervised or semi-supervised phenotyping methods. Our example is based on MAP[28,36].

1. Extract the silver-standard labels and healthcare utilization feature for patients in data mart. We recommend using total days with disease PheCode and total number of disease CUI as silver-standard labels and total days with any ICD code as healthcare utilization. Our recommendation stems from the observation that multiple codes in one day merely reflect the administration pattern (less for integrated provider and more for segmented providers) yet multiple mentions of the disease in medical notes usually indicate likelihood of disease onset. Manual chart review to obtain the gold-standard

labels for a random subset, e. g. 59 patients as in Module 4, should also be done in parallel.

2. Apply an unsupervised phenotyping method, e. g., MAP, and validate the performance with the gold-standard labels. If the numeric prediction is reasonable (area-under-receiver-operating-characteristic-curve, AUROC >0.9), choose the threshold with 0.95 specificity and construct the disease cohort with patients whose numeric prediction is greater than the threshold. Otherwise, go to next step.
3. Extract EHR variables from the expanded mapping and generate additional gold-standard labels, e.g., 200 patients. Run a semi-supervised phenotyping method, e.g., PheCAP.

For phenotyping of colorectal cancer patients, the silver standard labels can be the diagnosis codes under PheCode 153 and the main "colorectal cancer" CUI C0009402. The healthcare utilization can be measured by the number of days with ICD codes. We used the total counts (across all encounters) of these three features as inputs to MAP algorithm.

<u>Create the treatment arms.</u> Most medication or procedure based treatments are mapped to structured EHR codes. With the mapping established in Module 1, we can create treatment arms of patients with the correspondent treatment code. Patients with colectomy or laparoscopy-assisted colectomy current procedure terminology (CPT) codes formed the open colectomy arm and the laparoscopy-assisted colectomy arm [28]. The list of codes was identified by "partial colectomy" in code descriptions:

1. Open colectomy: C44140, C44145, C44146, C44160.
2. Laparoscopy-assisted colectomy: C44204, C44205, C44207, C44208

The creation of treatment arms in accordance with indication of target RCT may involve ascertaining the temporal order of disease onset/progression and the treatment. For COST

Study Group Trials, we set the date of first partial colectomy code as treatment initiation date and matched the indications by the following rules:

1. Patients with first colorectal cancer diagnosis code after treatment initiation date were excluded, as the surgeries were likely intended for treatment of other diseases.
2. Patients with multiple surgical codes around first colectomy CPT (3 days before to 3 days after) were excluded, as they likely underwent more complex procedures.
3. Patients were required to have a recent radiological test (42 days within registration) and undergo a colectomy within 21 days, so we interpreted the requirement as the implicit eligibility that a colectomy must be done within 90 days following the colorectal cancer diagnosis.

A further refinement of the treatment arms will be done in Module 3 with the curated data for eligibility criteria.

While the indication for COST Study Group Trial is relatively straightforward, indications for other studies may require delicate learning process. For example, the indication “first-line therapy for metastatic cancer” would be more challenging to identify because the indication allows prior therapy before metastasis as adjuvant therapy but excludes other therapies between metastasis and the therapies of interest. For such studies, the temporal phenotyping [37] would be necessary. The workflow includes the following steps:

a. Extract the encounter level EHR variables informative for treatments of interest and the disease onset/progression in indication. For each patient, create the marginal counts for each variable and the ordered counts for each pair (SPM and tSPM) [37].
b. Create the silver standard label to perform features screening and model selection. Order counts for treatment of interest variables after indication disease onset/progression variables are natural choices for the silver standard label. Other rule-based extraction can also be used here.

c. Train the temporal phenotyping model with selected features on gold-standard labels of expert annotated treatment and indication. The numeric probabilities are produced.
d. Determine the optimal threshold for numeric probability over another set of gold-standard labels with minimal miss-classification.

To identify first line therapy for metastatic colorectal cancer, some gold standard labels on the first line use of the targeted therapy or chemotherapy of interest. Silver standard labels may be constructed from ordered counts of any indicators for metastasis (NICE [38] extraction of metastasis or cancer staging, PheCode 198) before the indicators for therapies (NLP extraction of regimen and medications, medication codes). Marginal counts and ordered counts for variables mapped to "metastasis" and the list of targeted therapies or chemotherapies of interest.

Ascertain treatment initiation time. The time-zero is obtained as the date of first treatment indicator or the first *qualified* treatment indicator if temporal phenotyping is involved. For the emulation of COST Study Group Trial, the time-zero is the date of first colectomy or laparoscopy assisted colectomy code. For the first line therapy for metastatic cancer, the time-zero is the date of first code of targeted therapies or chemotherapies after indicator for metastasis.

Section S3. Details and example for Module 3.

We provide details on variables curation for the emulation of COST Study Group Trial.

Phenotype-derived baseline variables: Most phenotype-derived variables were comorbidities in exclusion criteria. Considering the diagnosis codes are usually sensitive but not very specific for disease diagnosis, we chose the strategy of excluding patients with any diagnosis code mapped to the exclusion criteria. The strategy will create a conservative eligible cohort that has little chance to violate the exclusion criteria. For prior cancers, adhesions, Crohn's disease, familial polyposis, and chronic ulcerative colitis, we searched for diagnosis codes between 5 years prior to the colectomy date and the colectomy date. For colon obstruction and perforation, we searched for diagnosis codes between 30 days prior to the colectomy date and the colectomy date.

NLP-derived baseline variables: For cancer stage, we extracted from natural language processing (NLP) of the medical notes between 1 years prior to the colectomy date and the colectomy date. We extracted cancer clinical stage using the NLP Interpreter for Cancer Extraction (NICE) tool [38]. In the Mass-General-Brigham healthcare EHR, clinical stages (e.g., "stage 1" or "stage IV") or more detailed tumor-node-metastasis (TMN) stages (e.g., T1N2M0) are commonly documented in medical notes for cancer patients. Among the colorectal cancer disease cohort, there are 65.2% with at least one mention of either clinical or TMN stages. We validated the extraction accuracy by 140 notes from the colorectal cancer data mart with annotated cancer stage mentions (79 with cancer stage information, 61 without cancer stage information). NICE achieved very high capture rate (no false negative extraction, 1 false positive extraction). The extractions of numerical stage for all 79 notes with cancer stage information were accurate.

Other baseline variables: A few surgery-related characteristics requires separate tools tailored for those variables. In the emulation of COST Study Group Trials, we encountered the following variables:

1. Location of the Tumor: We used the location specific diagnosis and surgical codes to identify the location of tumor.
   a. Right colon: ICD10 C18.0-3; ICD9 153.0, 153.4-6; CPT C44160, C44205.
   b. Left colon: ICD10 C18.5-6 ICD9 153.2, 153.7.
   c. Sigmoid colon: ICD10 C18.7, C19; ICD9 153.3, 154.0; CPT C44145, C44146, C44207, C44208.

   Patients with multiple locations according to the codes were excluded in accordance with the eligibility criterion regarding tumor in single segment of colon.

2. Size of the Tumor: We were unable to replicate the criterion for tumor size due to the absence of exact information in EHR medical reports. Although we found NICE to be very accurate in extraction of documented tumor sizes in radiology reports, there was no routine documentation of tumor size according to research standard like RECIST [39].

While we relied on the rule-based variable curation in the current example, applications of image artificial intelligence may provide detailed measures of tumor characteristics in future studies [40, 84].

Endpoint: The overall survival endpoint is defined by the time from treatment initiation to death. Death information is mostly available from linking patients in Mass-General-Brigham EHR to their records in the death registry of the Commonwealth of Massachusetts. To capture missing death records, we constructed a terminal status score at the end of EHR follow-up. The formula of the score is:

$$raw\ terminal\ score = \log(1 + \#last\ month\ diagnosis) + \log(1 + \#last\ month\ procedure),$$

$$terminal\ score = death\ record + (1 - death\ record) * \frac{raw\ terminal\ score}{maximum\ raw\ terminal\ score}.$$

Over 100 labels on terminal status annotated by a clinically trained abstractor, we observed 9 missing death records while the terminal score is highly predictive for death/terminal status at the end of follow-up with area-under-reception-operator-curve (AUC) 0.954. We selected the threshold as 0.5 to approximate the death/terminal status rate in the 100 labeled patients, i. e. the event status is set as 1 if the terminal score is larger than 0.5.

Section S4. Details and examples for Module 4

Validation sample size calculator

We derive the validation size calculator under two assumptions: 1) patient data is independent across individuals; 2) validation sample is a relatively small subset in the large study cohort. Under such setting, the number of errors detection among validation sample can be modeled by a binomial distribution with size being validation sample size and probability being overall error rate,

$$Detection\ Chance = P(number\ of\ erros \geq 1) = 1 - P(number\ of\ errors = 0)$$
$$= 1 - (1 - Error\ Rate)^{Validation\ Size}.$$

Solve the equation for validation size, we will get the formula,

$$Validation\ Size = \frac{\log(1 - Detection\ Chance)}{\log(1 - Error\ Rate)}.$$

We obtain the lower bound for validation size according to the following facts:

1. Fixing error rate, increasing validation size will result in higher detection chance. To ensure integer number for validation size, we relax the equation to inequality, which will guarantee a higher than nominal error detection chance,

$$Validation\ Size \geq \frac{\log(1 - Detection\ Chance)}{\log(1 - Error\ Rate)}.$$

2. The formula is monotone decreasing in error rate. Since the true error rate is unknown in practice, we set an error tolerance for the calculation of validation size that guarantees a higher than nominal error detection chance whenever true error rate is higher than the error tolerance ($Error\ Rate \geq Error\ Tolerance$),

$$Validation\ Size \geq \frac{\log(1 - Detection\ Chance)}{\log(1 - Error\ Tolerance)} \geq \frac{\log(1 - Detection\ Chance)}{\log(1 - Error\ Rate)}.$$

Efficient Sampling scheme

As annotations are needed at various places, from cohort construction through variable extraction to validation, a strategy to reuse them may maximize the utility of the time and labor put into annotation. To facilitate robust analysis, it is important to annotate all error-prone variables over the same subset for downstream analysis. Aiming for this goal, we propose the following sampling scheme.

1. Randomly shuffle the initial data mart. Whenever k annotations are needed during the cohort construction and variable extraction, always choose the first k qualified patients according to the post shuffling order.
2. Prioritize the extraction of eligibility criteria so that the extraction of other variables may be focused within the preliminary emulation cohort.

   The scheme maximizes the overlapping of the annotated sets for various tasks so that all annotations qualified at a later stage can be reused.

Robust adjustment for confounding

The statistical analysis is detailed in **Section S2,** Supplementary Materials of Hou et al. [28]. We accounted for confounding from:

1. Clinically relevant variables: age, gender, cancer stage, tumor location, colon adhesion, procedure subtypes, obesity curated from Module 3.
2. A broad range of co-morbidities represented by the overall and one-year re-treatment occurrence of diagnosis codes rolled up to PheCode integer level groupings.
3. Calendar year and its interaction with other variables.

We adopted a doubly robust causal modeling strategy under which we trained 1) the outcome regression (OR) model of overall survival by a Cox model; 2) the propensity score (PS) model by logistic regression models. To account for temporal changes for ATE estimation, we allowed the covariate effects in both the OR and PS models to vary across the temporal periods but adopted a co-training strategy allowing the data to determine the degree of similarity between the three sets of models across time periods. We used group adaptive least absolute shrinkage and selection operator (LASSO) estimation [41- 43] for both the OR and PS training to incorporate feature selection and high dimensionality of the confounders.
